# KIR4.1 Antibodies as Biomarkers in Multiple Sclerosis

**DOI:** 10.3389/fneur.2014.00062

**Published:** 2014-04-30

**Authors:** Marie Wunsch, Damiano M. Rovituso, Stefanie Kuerten

**Affiliations:** ^1^Department of Anatomy and Cell Biology, University of Wuerzburg, Wuerzburg, Germany

**Keywords:** CNS antigen, epiphenomenon, KIR4.1, parietal cells, MS

Multiple sclerosis (MS) is an autoimmune disease caused by the infiltration of autoreactive lymphocytes into the central nervous system (CNS). The influence of B cells has been underestimated for a long time. Recently, it has become more apparent that B cells can fundamentally contribute to the pathogenesis of MS in terms of antigen presentation, co-stimulation, cytokine production, ectopic lymphoneogenesis, and antibody secretion (1–3). Along these lines, B cell depletion in MS patients with a relapsing-remitting disease course was able to reduce inflammatory brain lesions and clinical relapses (4) and B cell activation influences T cell polarization in the animal model of MS (5). Great effort has been made to utilize B cells for dividing patients into subgroups and to predict treatment responses in individual patients to specific drugs (6). Especially the detection of autoantibodies against CNS antigens is a crucial research subject due to its capability to be used as disease-specific biomarkers for the diagnosis and prognosis of MS.

To this end, in a clinical study published in 2012 in the *New England Journal of Medicine*, Srivastava and colleagues screened serum samples aiming to identify CNS-specific antibodies in MS (7). The authors identified the glial potassium channel KIR4.1 as one of the serum antibody targets in MS patients. Subsequently, an enzyme-linked immunosorbent assay (ELISA) large-scale screening with 397 MS patients, 329 persons with other neurological diseases, and 59 healthy donors was performed. Antibodies against KIR4.1 were observed in 46.9% of patients with MS, but were essentially absent in people with other neurological diseases and healthy donors. Based on these data, Srivastava et al. concluded that KIR4.1 is a CNS-specific target of the autoantibody response in a subgroup of patients with MS.

We would like to carefully raise the possibility that the serum IgG reactivity against KIR4.1 might present an epiphenomenon and there could be an additional target for autoreactive B cells beyond the CNS. It has to be considered that KIR4.1 is not only expressed in the CNS, but also on parietal cells of the gastric mucosal epithelium (8). The KIR4.1 channel expression is essential for the parietal cell control of acid secretion (9, 10). It is well known that there is a connection between the gastrointestinal immune response and CNS autoimmunity (11, 12). There are several possible mechanisms including molecular mimicry, determinant spreading, or bystander activation explaining the link between gastrointestinal immune response and MS (13). As recently published by Banati et al. (11), antibodies against parietal cells are also present in the sera of patients with MS. The presence of anti-parietal cell antibodies was most frequently associated with gastrointestinal complaints of the MS patients (11). Thus, it is possible that an impaired gastric mucosa, which can appear in cases of a frequent corticosteroid therapy, could extensively expose the parietal cell KIR4.1 to B cells. Corticosteroids augment gastroduodenal permeability and high doses are associated with macroscopic mucosal lesions (14).

Therefore, we consider it as necessary to include the history of gastrointestinal complaints in the anamnesis of the study subjects before defining KIR4.1 as a CNS target of serum antibodies. In the setting of patients with an initial demyelinating event who have not received corticosteroids, one could readily exclude an impact of the MS medication on the gut. Finding anti-KIR4.1 antibodies in such patients in combination with gut impairments could raise the possibility that the gut itself is also a target of the autoimmune process.

While further investigations are awaited to clearly analyze the impact of immune responses against antigens of gastric parietal cells on the finding of KIR4.1-reactive antibodies in patients with MS, the diagnostic potential of serum KIR4.1 antibodies may be highly clinically relevant. Therefore, the sensitivity and specificity of the initial findings of serum KIR4.1 antibodies in a subgroup of MS patients should be confirmed in studies from independent researchers.

Furthermore, the pathogenicity of KIR4.1 antibodies needs to be clarified. Only 2 of 19 serum IgG positive MS patients also had detectable levels of KIR4.1 antibodies in the cerebrospinal fluid (CSF) (7). By analogy, aquaporin-4 antibodies were not readily detected in the CSF in neuromyelitis optica patients (15). Immunhistochemical investigations by Lennon et al. (16) showed that serum aquaporin-4 antibodies in patients with neuromyelitis optica bind to CNS tissue as well as to parietal cells of the gastric mucosa (16). However, it is beyond doubt that aquaporin-4 antibodies are highly pathogenic in neuromyelitis optica since they were studied extensively. Kinoshita and colleagues have demonstrated the pathogenic role of aquaporin-4 antibodies *in vivo* (17). They obtained serum IgG from patients with neuromyelitis optica and detected astrocytic damage in rats after passive transfer of aquaporin-4 containing IgG. *In vivo* experiments of this kind could delineate whether KIR4.1 antibodies are only a disease marker or if they play a central pathogenic role. To this end, KIR4.1 antibody containing serum could be injected into mice and subsequent histological and immunohistochemical investigations could show potential resulting pathological effects in CNS and gut. Furthermore, astrocytes should be incubated with KIR4.1 antibody containing serum and cell viability assays may demonstrate whether KIR4.1-reactive IgG induces astrocytic cytotoxicity.

## Conflict of Interest Statement

The authors declare that the research was conducted in the absence of any commercial or financial relationships that could be construed as a potential conflict of interest.
